# Editorials

**Published:** 1909-02

**Authors:** 


### EDITORIALS

The Business Office of the JOURNAL-RECORD is 1 1-2 to 5 1-2 South Broad Street.
The Editorial Office is 1014-15 Century Building.
Address all Business Communications to *Journal-Record of Medicine*, 1 1-2 to 5 1-2 South Broad Street.
Make remittances payable to *THE JOURNAL-RECORD OF MEDICINE.*
On matters pertaining to the Editorial and Original Communications, address Edgar G. Ballenger, M. D., Atlanta, Ga.
Reprints of Original Articles will be furnished at cost price. Requests for the same should always be made in THE MANUSCRIPT.
We will present, postpaid, on request, to each contributor of an original article, twenty (20) marked copies of THE JOURNAL-RECORD OF MEDICINE containing such article.

### DEATH OF DR. W. B. ARMSTRONG.

It is with a feeling of unusual sorrow that we chronicle the untimely death of Dr. William Buckingham Armstrong, one of Atlanta's most successful and loved young physicians. Dr. Armstrong died of pneumonia complicating chicken pox, February 2, at his home, aged 35. His death is a great loss to the medical profession of this city, and the Journal-Record of Medicine and its staff extends deepest sympathy to his bereaved relatives and friends.

Dr. Armstrong was born in Atlanta in 1874, and here he received his preliminary education; his collegiate course was taken at the University of Georgia, where he was graduated in 1894. He was a member of the Chi Phi fraternity. Like his illustrious father he determined upon medicine as his avocation and entered the College of Physicians and Surgeons, New York, from which school he was graduated, with honors, in the class of 1899. After several years service in the New York hospitals, Dr. Armstrong began the practice of medicine in Atlanta, where he met with notable success. He was soon made associate professor of Anatomy in the Atlanta College of Physicians and Surgeons, and was appointed upon the visiting staff of the Grady, Presbyterian and Wesley Memorial Hospitals; he was also serving upon the local board of health at the time of his death. Dr. Armstrong was a careful, conscientious worker and his services were held in high esteem by all of patients; he also took much interest in the scientific part of medicine and was a regular attendant and interested member of the Fulton County Medical Society and the Georgia Medical Association.

In 1901 he married Miss Ruby Dart, of Brunswick, Ga.; he left one son less than three years of age.

As worthy citizen, a lovable physician, a devoted father and husband, Dr. Armstrong's loss will be keenly felt, and in the hearts of us all his place will long be held dear.

---

### EXAMINATION OF SCHOOL CHILDREN.

---

Regarding the feasibility and advisability of making regular physical examination of all school children, the Committee of the Fulton County Medical Society appointed to make an investigation of the matter, made the following report which was unanimously adopted:

That all school children should be examined for the presence of contagious and other diseases.

That all examinations should be made by physicians, or under their direction.

That the examining physicians should be general practitioners of medicine, and not specialists.

That such examining physicians should be elected on competitive examination to be given by the medical members of the City Board of Health, and the President of the Fulton County Medical Society.

That the examining physicians should devote all their time to this work, not be allowed to do any private practice or treat any case whatsoever, shall accept no remuneration other than is here specified, or shall in no case advise concerning what physician shall treat any case.

That they be elected annually.

That the Examining Committee shall have power to reappoint the physicians annually if their services have been satisfactory.

That any examining physician, desiring to resign should give three months' notice.

That the examining physicians shall consist of the requisite number of physicians who shall be paid each, $1,200 per annum, and that they shall be under the control of a joint committee from the Board of Education and the Board of Health, with the health officer of the city as direct superior.

That the city medical officer make a complete report to the Board of Health once a month.

That each examiner be required to keep a card index system, showing the condition of each child's examination, and shall make a written report to the city medical officer.

That no report of the child's condition can be received unless the examination of the child has been made by the authorized examiner of the school where the child attends.

We believe that it would be inexpedient for the city physicians to undertake this work of examining the school children.

That a corps of nurses, sufficient for the needs of the service, should be employed to assist the physicians.

That these nurses should be selected in the same manner as the examining physicians. They shall not advise concerning what physician shall be consulted by any child.

That the teachers also, be examined annually, and shall also be under the supervision of the medical examiner.

That teachers should be included in the examination.

---

### GASTROPTOSIS IN TUBERCULAR PATIENTS.

---

The writer has recently read with much interest a paper in the South California Practitioner, in which Dr. Boardman Reed, of Los Angeles, and Dr. Frank Robinson, of Monrovia, discuss the question of gastroptosis in tubercular patients.

Just now, when tuberculosis in both its medical and sociologic aspects is being so keenly studied, any thoughtful expression from a competent observer will receive due weight.

In this paper there are 221 tubercular patients reported on, the time of observation extending over about fifteen months. Of this number, 108 were men and 113 women. Of these, 133 showing pronounced gastro-intestinal symptoms, their abdominal organs were carefully examined.

Out of the above 133 cases 50, or 37 per cent., showed more or less gastroptosis. Of these 50 cases, 28 were women and 22 men. Ten of them showed also movable right kidney, 9 being women.

This report would bring up the following questions: Was the presence of gastroptosis in such a number of coincidence; was it to any extent a causative factor; or did it follow in the wake of the tuberculosis?

Charles R. Stockton, in a paper appearing in 1900, writes as follows: "The fact that more than 50 per cent. of all civilized women in all classes of life have developed the condition known as enteroptosis, which means that the stomach and intestines, very often the kidneys, and sometimes the liver are dragged down and remain permanently out of their position, is not generally known. Such, however, is the case; and this condition more than any other cause is responsible for the constipation, backache, debility, biliousness, early loss of complexion, headache, and that long list of ailments of which so many women in all civilized countries are victims."

Einhorn, in 1901, reported on 1912 patients, 347, or 18 per cent. of which had splanchnoptosis including gastroptosis. Of these 70 were men and 277 women.

The writer's observation has led him to believe Stockton's estimate rather high for the present time, and Einhorn's rather low; the term *present time* being used advisedly, for the straight-front corsets now worn are certainly less liable to cause downward displacements than those of eight or ten years ago.

The conclusions of Drs. Reed and Robinson would indicate that the ptoses could only be regarded as predisposing factors. Many of the tubercular lesions were recent, while the ptoses showed evidences of long standing.

We can easly see that the disturbances of metabolism incident to gastroptosis would render the patient less able to resist the inroads of the infection. It would also naturally affect the system of forced feeding so heroically carried out by some; and would explain why some of the cases failed so utterly to respond to a generous diet.

The following is quoted from the paper mentioned, and is endorsed by the experience of the writer: "At all events our observation has proved that overcoming any coexisting visceral displacement has been followed by a better response to the essential curative measures for the principal disease."

"In all known tubercular cases, therefore, we should search for and correct any existing visceral displacement as a very helpful preliminary to the cure of the major disease; and to precent the development of tuberculosis in persons predisposed thereto by reason of lowered nutrition, the same precaution is equally important."

A careful examination of the abdomen, therefore, should never be omitted in these cases, for when a visceral displacement, especially a gastroptosis is found present, there will necessarily be a change in the dietetic and mechanical features of the general management of the patient.

G. M. N.

---

### THE PHYSICIAN AND BROUGHTON—EMMANUELISM

Much of the early history of medicine is intimately associated with the church, in fact during certain periods of the dark ages the church was the chief custodion of medical knowledge. Nearly 800 years ago, under Pope Calixtus II, the practice of medicine and the church were "forcibly rent asunder;" that we will again retrograde to a re-union seems exceedingly un-
we will again retrogarde to a re-union seems exceedingly unlikely. The emmanuelists are attempting to make such union, however, and purpose to use the physicians as tools for making diagnoses while they delegate to themselves the right to treat the patients, assuming superior knowledge in such matters. It does not seem unreasonable to take the ground that the doctor—knowing more about diseases—is legitimately entitled to carry out the treatment.

Psychotherapy has been an important method of treatment from time immemorial as medical literature shows; we have books devoted exclusively to this form of treatment and, while not heralding our results with it before the lay world any more than our cures of syphilis with mercury, it demonstrates our familiarity with such measures. It seems, now, rather preposterous for a few ministers, who have recently become interested in psychology to allow their enthusiasm to so exceed reasonable bounds that they consider themselves more competent to wield psychotherapy than physicians who are vastly more familiar with disease and, as a class, understand psychology equally as well or better than ministers We further think they are unjust in arrogating to themselves all of the "cheerfulness, hope, courage, faith and praise."

That some physicians are not as conversant with psychotherapy as they might be, may be granted—the same might be said, however of all therapeutic measures as well urinalysis and all diagnostic methods. The church can be a powerful agent in waging a warfare against disease by inculcating principles of right living, optimistic thinking, cleanliness and morality; in the actual treatment of disease itself the physician who makes it a special study is the one to whom such should be delegated.

### STATE SANITARIUM FOR CONSUMPTIVES.

Governor Smith has appointed the board of trustees for the state sanitarium for the treatment of tuberculosis and it is believed that the institution will soon be in operation.

At the last session of the general assembly an appropriation of $25.000 was made for the establishment of this institution, of which $1,000 was made available in 1908, and $12,000 in each of the succeeding years. This means there is now $13,000 available with which to begin the work, and the future of the project is now in the hands of the trustees.

The trustees may purchase a tract of land and provide for the erection of a building, or they may buy a building already erected and begin work at once. The details are entirely within their hands. It is probable that a meeting will be called at an early date and following organization a location will be secured.

Following is the board of trustees named by the governor consisting of two from each congressional district:

First Congressional District—Dr. P. S. Clarke, of McIntosh, and Hon. C. W. Skinner, of Burke.

Second District—Dr. E. Daniels, of Colquitt, and Dr. R. C. Woodward, of Berrien.

Third District—Dr. C. H. Richardson, of Macon county, and Dr. J. R. Statham, of Sumter.

Fourth District—Dr. H. R. Slack, of Troup, and Hon. W. B. Short, of Marion.

Fifth District—Hon. W. G. Raoul, of Fulton, and Dr. T. R. Whitley, of Douglas.

Sixth District—Hon. T. D. Tinsley, of Bibb,and Dr. M. F. Carson, of Spalding.

Seventh District—Dr. C. F. McLain, of Gordon, and Hon. J. D. Anderson, of Cobb.

Eighth District—Dr. W. I. Hailey, of Hart, and Hon. J. D. Harvey, of Jasper.

Ninth District—Dr. Jeff Davis, of Stephens, and Hon. M. S. Cornett, of Gwinnett.

Tenth District—Dr. W. B. Crawford, of Lincoln, and Hon. T. I. Hickman, of Richmond.

Eleventh District—Dr. J. A. Butts, of Glynn, and Dr. W. H. Born, of Telfair.

The terms of office of the appointees are four years and they are to have entire authority in the location, establishment and management of the new state institution.

